# Heterotopic Gastric Mucosa Presenting as Lower Gastrointestinal Bleeding: An Unusual Case Report

**DOI:** 10.1155/2019/5791984

**Published:** 2019-12-28

**Authors:** Syed Muhammad Ali, Ayman Abdelhafiz Ahmed, Leena Amin Hussain Saaid, Gihan Mustafa Kamal Mohamed, Amjad Ali Shah, Mohannad Al-Tarakji, Zia Aftab, Inamulla, Sameera Rashid

**Affiliations:** ^1^Department of Surgery, Hamad Medical Corporation, Doha, Qatar; ^2^Department of Colorectal Surgery, Hamad Medical Corporation, Doha, Qatar; ^3^Department of Pathology, Hamad Medical Corporation, Doha, Qatar

## Abstract

Heterotopic gastric mucosa (HGM) is gastric mucosal tissue outside the stomach. It can be discovered anywhere throughout the gastrointestinal tract and is mostly asymptomatic. HGM, although rare beyond the ligament of Treitz, should be included in the differential diagnosis in a young patient with a polyp causing obstructive symptoms or bleeding. Very few cases are published in literature. We describe a case of young male who presented with an episode of large amount of melena, from a bleeding jejunal lesion, diagnosed by endoscopy. Laparotomy and wedge resection of the jejunal lesion was done, and histopathology showed gastric heterotopia in a small jejunal diverticulum.

## 1. Introduction

Heterotopia (Greek origin), literally meaning different location, is the presence of a tissue type normally found in the human body but in an abnormal location [1] and can occur throughout the gastrointestinal tract. It can also involve the biliary tract, umbilicus, and even the scrotum [2]. Most of gastric mucosal heterotopias are nodules, polyps, or tumorous growths [3]. HGM of the intestinal tract is a gross or microscopic finding that is incidentally and occasionally detected at surgery or autopsy [4]. It can be asymptomatic or present with various complications like massive gastrointestinal bleeding and even death [5].

## 2. Case

A 23-year-old male was brought to the emergency of Hamad General Hospital in Doha, after being found on the floor dizzy and confused, with urinary incontinence although no one witnessed convulsions. The patient denied any fever, headache, chest pain, shortness of breath and palpitations, or cardiac diseases.

On examination, his pulse was 75 beats per minute, blood pressure 125/78, JVP was not raised, S1 and S2 were normal, and no added sounds on the chest examination. The patient was thought to have had a syncopal attack. His complete blood count and biochemical test were within the normal range. Computed tomography (CT) scan of the head was unremarkable and ECG showed left ventricular hypertrophy and early repolarization. Echocardiography, apart from moderate septal hypertrophy, was otherwise unremarkable.

Further history revealed that the patient had an episode of a large amount of melena before the syncopal attack, associated with nausea and two episodes of vomiting, but denied history of alcohol consumption, analgesic intake, and history of liver disease. In the course of time, the patient developed tachycardia of 122 bpm but blood pressure remained normal. His abdomen was soft, lax, nontender, and not distended. No masses were appreciated and bowel sounds were normal. Digital Rectal Examination (DRE) showed melanotic stool. Laboratory results showed a drop in hemoglobin from 13.2 to 8.4 and a high urea of 7.4. He was kept on intravenous fluids and proton pump inhibitors and transfused 2 units of packed red blood cells, and his hemoglobin level was monitored.

**Figure 1:**
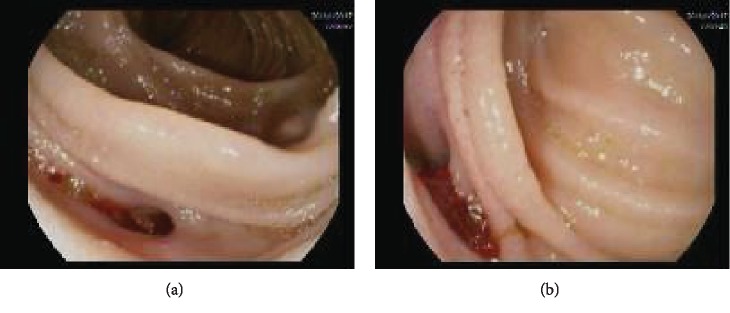
Enteroscopy view showing diverticulum in the jejunal fold with small amount of blood.

Esophagogastroduodenoscopy (EGD) showed normal examination up to the third part of the duodenum with no active bleeding or altered blood. Colonoscopy was normal, whereas enteroscopy showed a jejunal diverticulum about 50 cms from the pylorus with fresh bleeding (Figure 1). Patient remained hemodynamically stable with no further drop in hemoglobin or another episode of melena. CT angiography was done in an attempt to identify a source of bleeding and for a trial of coil embolization, but it did not reveal vascular blush into the small bowel but an incidental finding of horseshoe kidney.

The patient underwent diagnostic laparoscopy that did not show any abnormality in the jejunal loops externally. Endoscopy was carried out to localize the jejunal lesion, but it was difficult to pass the scope beyond the duodenojejunal (DJ) junction. Midline laparotomy was performed to guide the enteroscope to the upper jejunum. A small lesion was seen on the serosal surface of the jejunum 30 cm from the DJ junction which endoscopically appeared as a suspicious diverticulum with whitish mucosal plaques. There was no active bleeding or clots. The enteroscope was then pushed further up to 130 cm in the bowel but no pathology was observed. Wedge resection of the suspicious jejunal lesion was carried out. The patient made smooth recovery from the surgery and did not develop further episodes of bleeding. He was last seen in the clinic after 2 years of surgery without any symptoms.

**Figure 2:**
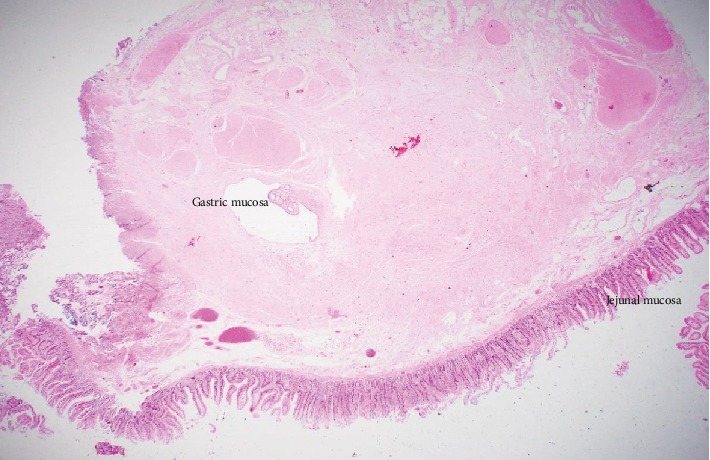
Low microphotograph of the jejunum showing an island of gastric mucosa in the center.

**Figure 3:**
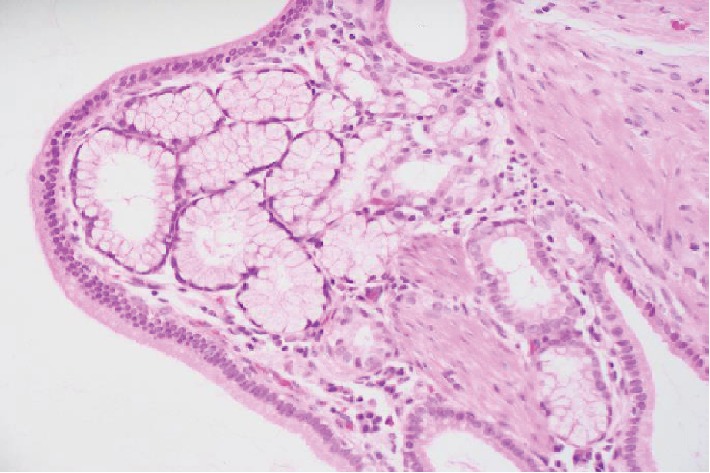
High-power microphotograph of the gastric mucosa with submucosal glands.

Histology showed a small jejunal diverticulum with gastric heterotopic tissue. The specimen consists of a false diverticulum of the small bowel (jejunum) showing a focal, cystically dilated gland within the muscularis propria which, on deeper sections, showed gastric-type mucosal tissue. Focal crypt rupture was noted with mild reaction. The mucosa was unremarkable, and the specimen was negative for dysplasia and malignancy (Figures 2 and 3)

## 3. Discussion

Gastric heterotopia is an uncommon condition, and it is important to differentiate it from metaplasia. Metaplasia is a change of one type of fully differentiated tissue to another, usually due to sustained inflammation. Heterotopias are developmental anomalies, whereas metaplasia is an acquired condition [2].

Gastric heterotopia has been reported anywhere throughout the length of the gastrointestinal tract, from the oral cavity to the anal canal. It can be congenital as seen in Meckel's diverticulum or intestinal duplication, although it is rare beyond the ligament of Treitz [6]. Salivary glands, biliary ducts and gallbladder, umbilicus and respiratory tract, bronchogenic and thyroglossal cysts, the spinal column, intra-abdominal and intrathoracic locations, the urinary bladder, and scrotum are other unusual sites reported for the presence of HGM [7].

HGM consists of full thickness of specialized gastric gland mucosa, containing chief and parietal cells and lined by a single layer of a simple columnar epithelium that is taller than its width (foveolar epithelium). The peptic secretion of these gastric glands is the cause of the intestinal ulcerations observed in cases of HGM [2, 8].

Clinical presentation of HGM varies and depends on the location and size of the heterotopic tissue. It usually presents as an inlet patch in the proximal esophagus, as polypoid masses in the rectum, or as nodular tumors in the duodenum [9]. HGM presenting as a mass in the jejunum causing gastrointestinal bleeding is very rare, like in our case, with very few reports published [10]. HGM can form an intraluminal mass and cause airway or intestinal obstruction depending on the site. It may also serve as the lead point for the intussusception. The most common complication of HGM is intestinal mucosal ulceration with GI bleeding. Intestinal perforation and fistula formation to adjacent structures have also been reported [6, 11].

The incidence of HGM in the esophagus varies widely from 0.1 to 13.8% and that in the duodenum from 0.5 to 8.9%. Gastric heterotopias are mostly seen in the duodenum but very rarely in the small intestines, as in our case. [9]

The mechanism for the development of HGM is still not completely elucidated. There are different theories used to explain the etiology [5]. During the process of developmental descent of the stomach, remnant tissue could have remained in the distal esophagus. However, this mechanism could explain only gastric heterotopia of the esophagus, not that in the more distal intestinal tract [9]. It may occur due to Cdx2 modifying the expression of molecules. Cdx2 stimulates markers of enterocyte differentiation. Null mutation of Cdx2 leads to the development of ectopic lesions with a gastric phenotype in the midgut endoderm [5].

Some authors have proposed that HGM is the result of an abnormal regenerative process following destruction of normal intestinal mucosa. This hypothesis is not supported by any descriptions of gastric heterotopia following destruction of gastrointestinal mucosa resulting from gastroenteritis or other inflammatory conditions of the intestines [12]. The final and most probable hypothesis is that HGM is the result of abnormal differentiation of local tissue. An error in the differentiation of pluripotent primitive endoderm stem cells could lead to the gastric mucosa being present anywhere throughout the gastrointestinal tract, as these cells have the ability to differentiate into cell types of the gastrointestinal epithelium [5, 9].

History-taking and physical examination are important, as they help to suspect the potential diseases involved. Radiologic investigation such as a CT scan, fluoroscopy, capsule endoscopy, and 99mTc pertechnetate scan can usually detect HGM [5]. Capsule endoscopies have been shown to be superior in the recognition of small bowel pathology when set side by side with other radiological investigations, including barium contrast studies, computed tomographic enteroclysis, push enteroscopy, and MRI, as capsule endoscopies have a positive diagnosis rate ranging from 45 to 76% [1].

The definitive diagnosis in all reported cases of HGM in the small intestine was established by histopathological examination of the surgically removed specimens, and surgery was performed for acute complications such as GI hemorrhage or perforation in the majority of cases [5, 11].

Surgery, including resection of the lesion and anastomosis of the healthy bowel ends, has been considered as the treatment of choice whether it is carried out by laparoscopy or laparotomy [6].

## 4. Conclusion

HGM is a rare condition and should be considered in the differential diagnosis of upper or lower gastrointestinal bleeding. Diagnosis may be difficult preoperatively but surgical resection offers cure.
